# Safety and efficacy of catheter ablation of atrial fibrillation in the very elderly (≥80 years old): Insights from the UC San Diego AF Ablation Registry

**DOI:** 10.1002/clc.24137

**Published:** 2023-08-25

**Authors:** Omar M. Aldaas, Douglas Darden, Praneet S. Mylavarapu, Amer M. Aldaas, Frederick T. Han, Kurt S. Hoffmayer, David Krummen, Gordon Ho, Farshad Raissi, Gregory K. Feld, Jonathan C. Hsu

**Affiliations:** ^1^ Division of Cardiac Electrophysiology at the University of California San Diego Health System La Jolla California USA; ^2^ A. T. Still University School of Osteopathic Medicine Mesa Arizona USA

**Keywords:** atrial fibrillation, catheter ablation, complications, hospitalizations, mortality, very elderly

## Abstract

**Background:**

Catheter ablation improves outcomes in symptomatic atrial fibrillation (AF) patients. However, its safety and efficacy in the very elderly (≥80 years old) is not well described.

**Hypothesis:**

Ablation of AF in the very elderly is safe and effective.

**Methods:**

We performed a retrospective study of all patients who underwent catheter ablation enrolled in the University of California, San Diego AF Ablation Registry. The primary outcome was freedom from atrial arrhythmias on or off antiarrhythmic drugs (AADs).

**Results:**

Of 847 patients, 42 (5.0%) were 80 years of age or greater with a median age of 81.5 (80–82.3) and 805 (95.0%) were less than 80 years of age with a median age of 64.4 (57.6–70.2). Among those who were ≥80 years old, 29 were undergoing de novo ablation (69.0%), whereas in the younger cohort, 518 (64.5%) were undergoing de novo ablation (*p* = .548). There were no statistically significant differences in fluoroscopy (*p* = .406) or total procedure times (*p* = .076), AAD use (*p* = .611), or procedural complications (*p* = .500) between groups. After multivariable adjustment, there were no statistically significant differences in recurrence of any atrial arrhythmias on or off AAD (adjusted hazard ratio [AHR]: 0.75; 95% confidence interval [CI]: 0.45–1.23; *p* = .252), all‐cause hospitalizations (AHR: 0.86; 95% CI: 0.46–1.60; *p* = .626), or all‐cause mortality (AHR: 4.48; 95% CI: 0.59–34.07; *p* = .147) between the very elderly and the younger cohort.

**Conclusion:**

In this registry analysis, catheter ablation of AF appears similarly effective and safe in patients 80 years or older when compared to a younger cohort.

## Graphical abstract

Kaplan–Meier plots of long‐term recurrence of atrial arrhythmias on or off antiarrhythmic drugs (excluding a 3‐month postprocedural blanking period), long‐term rate of all‐cause hospitalizations, and long‐term rate of all‐cause mortality. Patients who were 80 years of age or older and a younger cohort are compared.

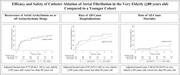

**Abbreviations**
AAD: antiarrhythmic drug
AF: atrial fibrillation
AFL: atrial flutter
AT: atrial tachycardia
CI: confidence interval
HR: hazard ratio

## 1 INTRODUCTION

Atrial fibrillation (AF) has become increasingly prevalent and its incidence increases with aging, with over half of AF patients projected to be over the age of 80 by 2050.^1^, ^2^ Catheter ablation has emerged as a viable alternative to long‐term pharmacologic antiarrhythmic therapy for rhythm control of AF and will likely be used more commonly given its inclusion in guidelines and increasing evidence in favor of an early rhythm control strategy.^3^, ^4^ However, the management of AF in the elderly is complicated by concurrent comorbidities, age‐related physiological changes, and frailty.^5^, ^6^ While there are multiple studies evaluating catheter ablation in the elderly, the majority of patients included were less than 70 years of age.^7^, ^8^, ^9^, ^10^ The safety and efficacy of catheter ablation in the very elderly (≥80 years old) population is thus not as well described.^11^

## 2 METHODS

This study was an observational, retrospective cohort study using data collected as part of the University of California (UC) San Diego AF Ablation Registry and approved by the UC San Diego Institutional Review Board. The UC San Diego AF Ablation Registry was designed as a clinical registry of all patients undergoing left atrial ablation procedures for atrial arrhythmias at UC San Diego, a single academic center, as captured by a procedural database (Perminova Inc.) to collect patient, provider, and intraprocedural characteristics. All AF ablation procedures captured by the registry from October 2009 to March 2015 were linked to clinical encounters as recorded by the electronic medical record at UC San Diego Medical Center (Epic). Data on baseline demographics, medical history, laboratory data, medications, and cardiovascular implantable devices were collected as part of the UC San Diego AF Ablation Registry. Intraprocedural registry reports were reviewed to determine fluoroscopy and procedure times and ablation lesion sets.

Patients were stratified into groups based on whether they were 80 years of age and older or younger than 80 years of age. Clinical outcomes were determined during all follow‐ups and included in‐hospital adverse events, recurrence of atrial arrhythmia at final follow‐up on or off antiarrhythmic drugs (AAD) and off AAD, all‐cause hospitalizations, and mortality. Arrhythmia recurrence was defined as AF, atrial flutter (AFL), or atrial tachycardia (AT) lasting ≥30 seconds recorded on a 12‐lead electrocardiogram (ECG), on ambulatory monitoring, or on implantable device interrogation, as recommended by contemporary guidelines.^12^ Patients who were continued on AAD after the 3‐month blanking period were censored from the analysis assessing recurrence of atrial arrhythmias off AAD.

Adverse events were recorded in the registry and included access site complications (e.g., bleeding, groin hematoma, pseudoaneurysm, and arteriovenous fistula), cardiac perforation or tamponade, stroke or transient ischemic attack, pericarditis, myocardial infarction, atrioesophageal fistula, phrenic nerve injury, and pulmonary vein stenosis.

As part of the registry, follow‐up arrhythmia monitoring was prespecified and was recommended as a 12‐lead ECG at each follow‐up visit, along with routine ambulatory ECG monitoring (24‐hour Holter monitor, extended ambulatory ECG monitoring, or event monitoring) in all patients at 6 months, 1 year, and 2 years after ablation and additional ambulatory ECG monitoring to evaluate for arrhythmia recurrence in the presence of suggestive symptoms, which was consistent with consensus guidelines and updated consensus guidelines at the time of the registry.^12^, ^13^

Informed consent was obtained before all ablation procedures. General anesthesia was used in all cases. Intravenous heparin was administered to target an activated clotting time of 300–400 seconds before and during left atrial access. A transseptal puncture was performed under direct visualization with intracardiac echocardiography. Pulmonary vein isolation was performed using segmental, circumferential, or both types of ablations at the discretion of the operator. Closed and open irrigated and noncontact and contact force sensing catheters were used at the discretion of the operator. Electroanatomic mapping systems were used in all cases (CARTO^TM^, Biosense‐Webster Inc. or Ensite^TM^, St. Jude Medical Inc.). Pulmonary vein entrance and exit block were confirmed with the use of a circular catheter, and adenosine and isoproterenol were administered at the operator's discretion. Additional lesion sets including a cavotricuspid isthmus line, left atrial roof line, posterior mitral isthmus line or anterior line, coronary sinus ablation, and ablation of complex fractional atrial electrograms were performed at the discretion of the operator.

Continuous variables are presented by group as means ± 1 standard deviation for normally distributed variables and as medians with 25th and 75th percentiles for variables that were not normally distributed. Comparison between all groups was done using the nonparametric Kruskal–Wallis tests. All possible comparisons among groups were performed using the Student *t*‐test if the data were normally distributed or the Wilcoxon rank‐sum test if the data were not normally distributed. Categorical variables were reported as count and percentage, with the *χ* ^2^ or Fisher exact test (expected cell counts < 5) used for comparisons.

Recurrence of atrial arrhythmias at the final follow‐up was analyzed using the Kaplan–Meier method with a 3‐month blanking period and log‐rank significance testing. Unadjusted and adjusted Cox proportional hazards modeling was used to analyze recurrence of atrial arrhythmias with a 3‐month blanking period and results are presented as hazard ratios (HRs) with 95% confidence intervals (CIs). Patients who were lost to follow‐up were censored at the date of last known follow‐up. Covariates included in the adjusted model are presented in Table 1, which were selected based on a clinically plausible association of the predictor variable with recurrence of the primary outcome of recurrent atrial arrhythmias. Missing values were minimal and roughly equivalent between groups for all variables and were thus omitted. Analyses were performed using Stata 11 (StataCorp LLC) statistical software. A *p* < .05 was considered statistically significant.

**Table 1:** Baseline characteristics.

	Very elderly (≥80 years old) (*n* = 42)	Less than 80 years old (*n* = 805)	*p* Value
Follow‐up duration (months)	42.3 (15.2, 61.5)	32.7 (10.0, 56.7)	.196
Age (years)	81.5 (80.6, 82.3)	64.4 (57.6, 70.2)	<.001
Male	19 (45.2)	230 (28.6)	.021
BMI (kg/m^2^)	24.4 (21.6, 28.0)	28.0 (25.2, 31.6)	<.001
De novo ablation	29 (69.0)	518 (64.3)	.548
AF type			.176
Paroxysmal	25 (59.5)	550 (68.3)	
Persistent	17 (40.5)	242 (30.1)	
CHA_2_DS_2_VASc	4.0 (3.0, 5.0)	2.0 (1.0, 3.0)	<.001
Comorbidities			
CHF	11 (26.2)	118 (14.6)	.071
HTN	32 (76.2)	438 (54.4)	.007
HLD	22 (52.4)	314 (39.0)	.093
DM	3 (7.1)	83 (10.3)	.499
COPD	3 (7.1)	24 (3.0)	.138
OSA	4 (9.5)	98 (12.2)	.594
Prior CVA	5 (11.9)	69 (8.6)	.464
CAD	12 (28.6)	130 (16.1)	.038
ESRD	0 (0.0)	4 (0.5)	.647
Smoker	7 (16.7)	137 (17.0)	.922
Echocardiographic parameters			
LVEF (%)	63 (56, 68)	62 (55, 67)	.508
LAD (cm)	4.22 ± 0.52	4.17 ± 0.64	.709
LVEDD (cm)	4.46 ± 0.67	4.85 ± 0.62	.013
MVR	13 (31.0)	153 (19.0)	.009
Cardiovascular medications			
Beta‐blocker	27 (64.3)	386 (48.0)	.042
Calcium channel blocker	12 (28.6)	221 (27.5)	.890
ACE‐I	8 (19.0)	150 (18.6)	.946
ARB	10 (23.8)	130 (16.1)	.193
Aldosterone antagonist	0 (0.0)	26 (3.2)	.237
Digoxin	5 (11.9)	72 (8.9)	.524
Aspirin	15 (35.7)	309 (38.4)	.705
Theinopyridine	1 (2.4)	21 (2.6)	.923
Coumadin	21 (50.0)	351 (43.6)	.436
Apixaban	1 (2.4)	36 (4.5)	.427
Rivaroxaban	4 (9.5)	122 (15.2)	.311
Dabigatran	5 (11.9)	94 (11.7)	.976
AAD preablation			
None			
Flecainide	3 (7.1)	139 (17.3)	.084
Propafenone	2 (4.8)	53 (6.6)	.634
Sotalol	14 (33.3)	174 (21.6)	.079
Dronedarone	2 (4.8)	76 (9.4)	.302
Amiodarone	7 (16.7)	88 (10.9)	.258
Dofetillide	1 (2.4)	24 (3.0)	.818
Device preablation			
PPM	5 (11.9)	49 (6.1)	.137
ICD or CRT‐D	0 (0.0)	28 (3.5)	.217

*Note*: Values are presented as median (Q1, Q3) for continuous variables or *n* (%) for categorical variables.

Abbreviations: AAD, antiarrhythmic drug; ACE, angiotensin receptor blocker; AF, atrial fibrillation; ARB, angiotensin receptor blocker; BMI, body mass index; CAD, coronary artery disease; CHA_2_DS_2_VASc, risk score for thromboembolic events; CHF, congestive heart failure; COPD, chronic obstructive pulmonary disease; CRT‐D, cardiac resynchronization therapy defibrillator; CVA, cerebrovascular accident; DM, diabetes mellitus; ESRD, end‐stage renal disease; HDL, high‐density lipoprotein; HF, heart failure; HFpEF, heart failure with preserved ejection fraction; HFrEF, heart failure with reduced ejection fraction; HTN, hypertension; ICD, implantable cardiac defibrillator; LAD, left atrial diameter; LVEDD, left ventricular end‐diastolic diameter; LVEF, left ventricular ejection fraction; MVR, mitral valve replacement; PPM, permanent pacemaker.

## 3 RESULTS

A total of 847 patients underwent catheter ablation during the study period with baseline characteristics summarized in Table 1. Of the analyzed cohort, 5.0% (*n* = 42) were 80 years of age or older, and among these patients, 29 (69.0%) were undergoing de novo ablation compared to 518 (64.3%) in the control group (*p* = .548). Median (Q1, Q3) follow‐up duration was 42.3 (15.2, 61.5) months in the very elderly group and 32.7 (10.0, 56.7) months in the control group (*p* = .196). The very elderly group had a higher proportion of males (45.2% vs. 28.6%; *p* = .021), lower body mass indices [24.4 (21.6, 28.0) vs. 28.0 (25.2, 31.6); *p* < .001], and more hypertension (76.2% vs. 54.4%; *p* = .007) and coronary artery disease (28.6% vs. 16.1%; *p* = .038). Additionally, the very elderly cohort was more likely to be prescribed a beta‐blocker (64.3% vs. 48.0%; *p* = .042). Ablation characteristics are summarized in Table 2. There were no significant differences in procedure times, fluoroscopy times, or additional ablation lesion sets between groups.

**Table 2:** Comparison of ablation characteristics and complications.

	Very elderly (≥80 years old) (*n* = 42)	Less than 80 years old (*n* = 805)	*p* Value
Total procedure time (minutes)	256 (211, 296)	236 (195, 282)	.076
Total fluoroscopy time (minutes)	78 (56, 95)	69 (54, 88)	.406
Additional ablation			
Mitral isthmus line	12 (28.6)	162 (20.1)	.204
LA roof line	13 (31.0)	239 (29.7)	.907
CFAE ablation	2 (4.8)	29 (3.6)	.711
CTI ablation	30 (71.4)	586 (72.8)	.754
Procedural complications			
Access site complication^a^			
Access site bleeding	2 (4.8)	62 (7.7)	.479
Groin hematoma	1 (2.4)	32 (4.0)	.600
Groin pseudoaneurysm	0 (0.0)	6 (0.7)	.574
Groin arteriovenous fistula	0 (0.0)	1 (0.0)	.819
Cardiac perforation/tamponade	0 (0.0)	3 (0.4)	.691
Stroke/TIA	1 (2.4)	3 (0.4)	.065
Pericarditis	1 (2.4)	5 (0.6)	.186
Other complications^b^			
Myocardial infarction	0 (0.0)	0 (0.0)	NA
Atrioesophageal fistula	0 (0.0)	0 (0.0)	NA
Phrenic nerve paralysis	0 (0.0)	0 (0.0)	NA
Pulmonary vein stenosis	0 (0.0)	2 (0.2)	.746

*Note*: Values are presented as median (Q1, Q3) for continuous variables or *n* (%) for categorical variables.

Abbreviations: CFAE, complex fractionated electrogram; CTI, cavotricuspid isthmus; LA, left atrial; TIA, transient ischemic attack.

^a^ Access site complications included access site bleeding, groin hematoma, groin pseudoaneurysm, and groin arteriovenous fistula.

^b^ Other complications included myocardial infarction, atrioesophageal fistula, phrenic nerve paralysis, and pulmonary vein stenosis.

**Figure 1:**
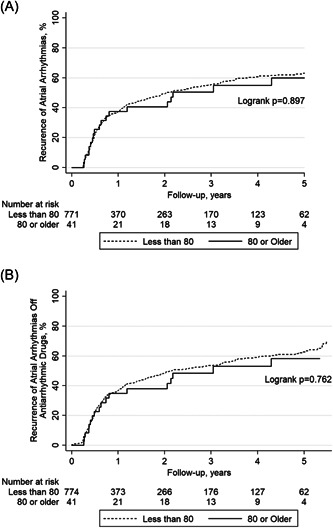
Kaplan–Meier plots of (A) long‐term recurrence of atrial arrhythmias (AF/AFL/AT) on or off antiarrhythmic drugs (excluding a 3‐month postprocedural blanking period), and (B) long‐term recurrence of atrial arrhythmias (AF/AFL/AT) off antiarrhythmic drugs. Patients who were 80 years of age or older and a younger cohort are compared. AAD, antiarrhythmic drug; AF, atrial fibrillation; AFL, atrial flutter; AT, atrial tachycardia.

There were no statistically significant differences in prevalence of procedural complications between groups (Table 2). Recurrence of atrial arrhythmias on or off AAD (50.0% vs. 46.1%; log‐rank *p* = .897) and off AAD (47.6% vs. 45.6%; log‐rank *p* = .762) was statistically similar in the very elderly compared to the younger control group during follow‐up (Figure 1A,B). Patients were on AAD after the 3‐month blanking period in 17 (40.5%) of the very elderly and in 360 (44.7%) patients in the younger control group (*p* = .126). There was no statistically significant difference in the frequency of patients undergoing repeat ablation in the very elderly group compared to the younger control group (26.2% vs. 24.8%; *p* = .819).

**Figure 2:**
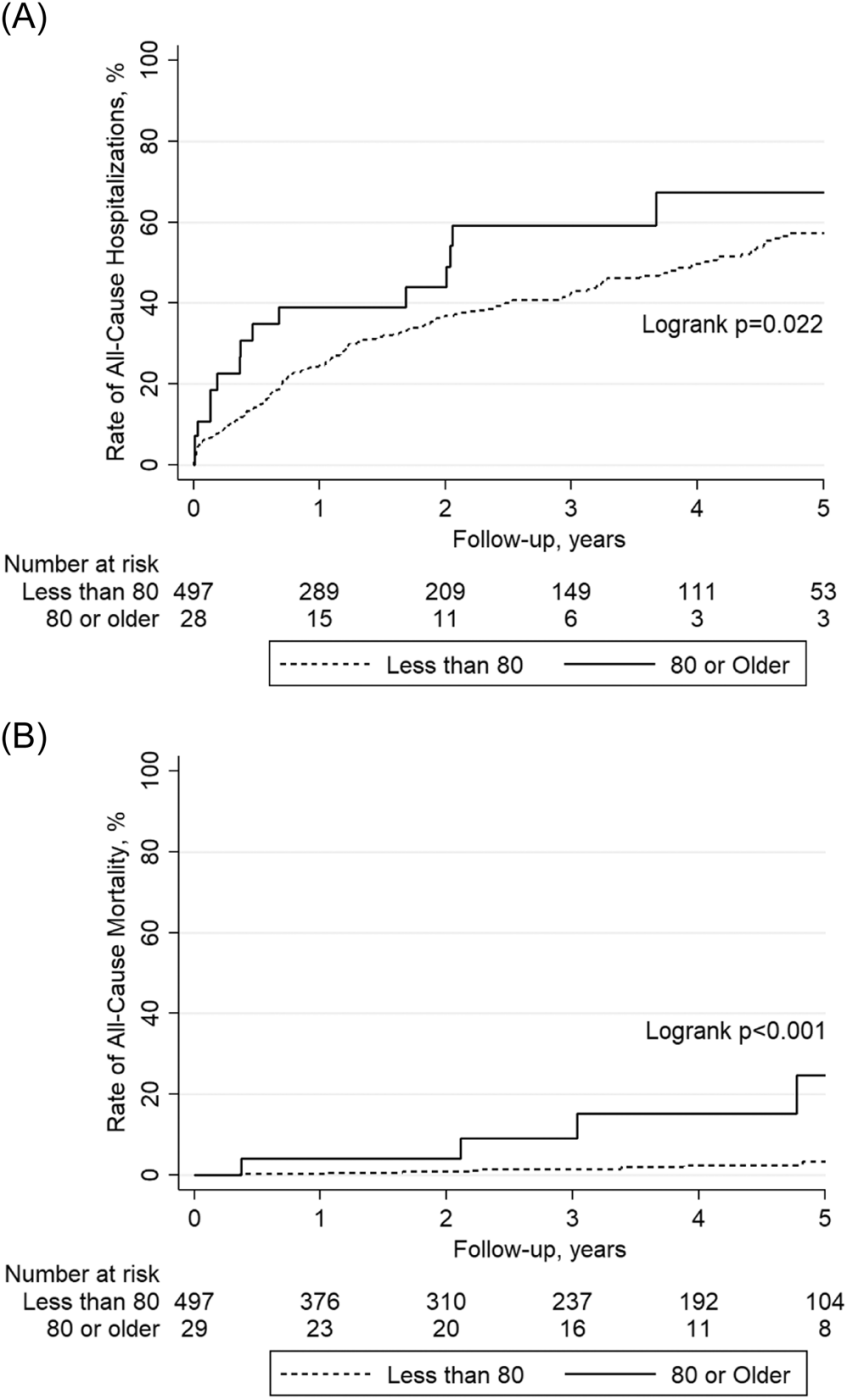
Kaplan–Meier plots of (A) long‐term rate of all‐cause hospitalizations and (B) long‐term rate of all‐cause mortality. Patients who were 80 years of age or older and a younger cohort are compared.

Unadjusted rates of all‐cause hospitalizations (42.9% vs. 26.6%; log‐rank *p* = .022) and all‐cause mortality (14.2% vs. 1.6%; log‐rank *p* < .001) were higher in the very elderly group compared to the younger control group during follow‐up (Figure 2A,B). However, this was no longer the case after multivariable adjustment. HRs with multivariable adjustment for potential confounders and respective CIs for recurrence of atrial arrhythmias and all‐cause hospitalizations and mortality are summarized in Table 3.

**Table 3:** Adjusted HRs and CIs.

Adjusted HR [very elderly (≥80 years old) vs. less than 80 years old]	*p* Value
Adjusted hazard of AF/AFL/AT on or off AAD	
0.72 (0.44–1.18)	.192
Adjusted hazard of AF/AFL/AT off AAD	
0.68 (0.41–1.12)	.133
Adjusted hazard of all‐cause hospitalizations	
0.92 (0.51–1.66)	.783
Adjusted hazard of all‐cause mortality	
2.48 (0.30–20.67)	.401

Abbreviations: AAD, antiarrhythmic drug; AF, atrial fibrillation; AFL, atrial flutter; AT, atrial tachycardia; CI, confidence interval; HR, hazard ratio.

## 4 DISCUSSION

In this retrospective cohort study, catheter ablation of AF appears to be similarly effective and safe in patients 80 years of age or older when compared to a younger cohort. The very elderly (≥80 years old) was selected as the study group as they were previously underrepresented in studies of catheter ablation of AF. Four previously published, small, observational studies reported similar complication rates between 2.5% and 7.5% for elderly patients, but did not evaluate differences in mortality.^7^, ^8^, ^9^, ^10^ Although another previous study evaluated the safety of catheter ablation in this population, with data reported on both procedural complications and mortality,^11^ it did not report data on the efficacy of catheter ablation in preventing recurrence of AF in this patient population. In this study, recurrence rates of atrial arrhythmias both off and on or off AADs were similar between groups over 5 years of follow‐up. Similar to what was previously published, rates of all‐cause hospitalizations and mortality in this study were also not significantly different between groups after multivariable adjustment.

The presence of more comorbidities in the very elderly group, such as coronary artery disease and hypertension, argues against selection bias. However, frailty is not taken into account as it is difficult to objectively quantify and it could thus be argued that the least frail, very elderly patients were chosen for a catheter ablation strategy.

The current analysis adds more to pre‐existing literature as it evaluated in‐hospital and long‐term outcomes on both the safety and efficacy of catheter ablation in the very elderly (≥80 years old) population compared to a younger cohort. Despite the age difference, there was no statistically significant difference in follow‐up between groups, with a median follow‐up of over 3.5 years in the very elderly group. Rhythm control with an ablation strategy is thus feasible in select elderly patients and there should not be an age limit above which patients are not considered for ablation. However, the median age of the very elderly group was 81.5, so it remains unanswered how safe and effective ablation of AF is in an even older population. These findings are significant as AF is the most common arrhythmia in the elderly, with the prevalence expected to increase over the next several decades.^14^ Although large studies have previously been published on the safety and efficacy of AF in elderly cohorts, the long‐term follow‐up involving outcomes such as atrial arrhythmia recurrence, hospitalizations, and mortality compared to a younger cohort makes these findings valuable.^15^, ^16^

Given a subset of patients remain symptomatic despite rate control, a rhythm control strategy is often pursued, especially since the higher rates of comorbidities present in the elderly population can make the use of AADs challenging. Furthermore, catheter ablation has been shown to be more effective in maintaining sinus rhythm and improving functional status and quality of life in the elderly compared to AADs.^10^ Despite these advantages, catheter ablation of AF is often not considered as a viable alternative in the elderly due to concerns for safety and efficacy, which is largely due to the lack of data in this population.

Although catheter ablation does carry an increased risk of embolization, uninterrupted anticoagulation has been shown to reduce thromboembolic events to less than 1% without increasing bleeding complications.^17^ However, there is currently little data on the safety and efficacy of uninterrupted anticoagulation during catheter ablation of AF in elderly patients. Still, this study and previously published studies have shown that anticoagulants, both vitamin K antagonists and direct oral anticoagulants, can be prescribed in the periprocedural period without significantly increasing bleeding risk.^18^

### 4.1 Study limitations

There are some limitations to interpreting the data presented in this study. First, the generalizability may be limited given that this study involved a single center and was a retrospective analysis. Second, the very elderly (≥80 years old) group was small in number relative to the control group. Third, it could be argued that very elderly (≥80 years old) patients selected to undergo catheter ablation were relatively healthier than the average patient in that age group; however, multivariable modeling was performed to account for differences in groups. Fourth, there was no standardized duration of monitoring for AF recurrence as it was left to the discretion of the clinician. However, at the minimum, guideline‐based recommendations were followed in all cases.^12^ Fifth, a significant percentage of patients were lost to follow‐up after 2–3 years.

## 5 CONCLUSION

Catheter ablation of AF appears to be similarly effective and safe in patients 80 years of age or older when compared to a younger cohort. There were no significant differences in all‐cause hospitalizations, mortality, or rates of recurrence of atrial arrhythmias.

## CONFLICT OF INTEREST STATEMENT

Dr. Hsu reports receiving honoraria from Medtronic, Abbott, Boston Scientific, Biotronik, Janssen Pharmaceuticals, Bristol‐Myers Squibb, Pfizer, Sanofi, Zoll Medical, Hillrom, iRhythm, Acutus Medical, and Biosense‐Webster, equity interest in Vektor Medical, research grants from Biotronik and Biosense‐Webster, and research funding support from the Marouf Family and the Butler and Gratt family. Dr. Ho reports receiving a research grant from Abbott, equity in Vektor Medical, and fellowship support from Medtronic, Abbott, Boston Scientific, and Biotronik. Dr. Feld reports receiving fellowship training program stipends (as CCEP fellowship training program director) from Medtronic, Biotronik, Biosense Webster, and Abbott Medical, has equity interest in Vektor Medical, is co‐founder and co‐owner of Perminova, and is a consultant to Acutus Medical. Dr Han receives research support from Abbott and honoraria from Abbott. Dr. Aldaas, Dr. Darden, Dr. Mylavarapu, Amer Aldaas, Dr. Hoffmayer, Dr. Krummen, and Dr. Raissi have no conflicts of interest to disclose

## Data Availability

There is no shared data available in this manuscript.
